# Underwater Holographic Sensor for Plankton Studies In Situ including Accompanying Measurements

**DOI:** 10.3390/s21144863

**Published:** 2021-07-17

**Authors:** Victor Dyomin, Alexandra Davydova, Igor Polovtsev, Alexey Olshukov, Nikolay Kirillov, Sergey Davydov

**Affiliations:** 1 Laboratory for Radiophysical and Optical Methods of Environmental Research, Faculty of Radiophysics, National Research Tomsk State University, 36 Lenin Avenue, 634050 Tomsk, Russia; dyomin@mail.tsu.ru (V.D.); polovcev_i@mail.ru (I.P.); asolshukov@gmail.com (A.O.); kns68@mail.tsu.ru (N.K.); 8952889579o@gmail.com (S.D.); 2 Laboratory for Remote Sensing of the Environment, V.E. Zuev Institute of Atmospheric Optics of the Siberian Branch of the Russian Academy of Sciences, 1 Academician Zuev Square, 634055 Tomsk, Russia

**Keywords:** underwater holographic sensor, plankton, ecosystem monitoring, accompanying measurements

## Abstract

The paper presents an underwater holographic sensor to study marine particles—a miniDHC digital holographic camera, which may be used as part of a hydrobiological probe for accompanying (background) measurements. The results of field measurements of plankton are given and interpreted, their verification is performed. Errors of measurements and classification of plankton particles are estimated. MiniDHC allows measurement of the following set of background data, which is confirmed by field tests: plankton concentration, average size and size dispersion of individuals, particle size distribution, including on major taxa, as well as water turbidity and suspension statistics. Version of constructing measuring systems based on modern carriers of operational oceanography for the purpose of ecological diagnostics of the world ocean using autochthonous plankton are discussed. The results of field measurements of plankton using miniDHC as part of a hydrobiological probe are presented and interpreted, and their verification is carried out. The results of comparing the data on the concentration of individual taxa obtained using miniDHC with the data obtained by the traditional method using plankton catching with a net showed a difference of no more than 23%. The article also contains recommendations for expanding the potential of miniDHC, its purpose indicators, and improving metrological characteristics.

## 1. Introduction

Modern marine science draws huge attention to global measurements both for fundamental purposes—building models of global ecosystems, and for solving applied problems, especially in predicting the efficiency and consequences of economic activities. This usually covers biological objects, primarily plankton [1], as the base of the global food chain and a natural bioindicator.

The study of marine suspended particles is not only related to the study and monitoring of plankton. Dredging and dumping of soil are carried out in large volumes during various hydraulic works in the shelf area (laying of underwater oil and gas pipelines, construction of port facilities, coastal bases and transshipment terminals, alluvion of territories). Meanwhile, part of the soil gets into water, goes suspended and may run to great, about hundreds of kilometers, distances as sedimenting non-living particles. Besides, oil droplets may be present in water in case of small leaks, for example, in the waters of an oil platform, oil pipeline, gas bubbles in the gas pipeline area, etc. These data are quite necessary for sustainable ecosystem observations in the water area of economic activity [2].

There are two types of ocean measurements-ecosystem monitoring and biodiversity monitoring of biological objects.

Ecosystem monitoring implies statistical analysis, and correlation in the trends of integral characteristics of representatives within the food chain or living conditions. Biodiversity monitoring involves detailed analysis, including systematic classification [3]. Ecosystem aspects may be analyzed by averaging the biodiversity data.

Some practical problems may only require integrated information obtained through ecosystem monitoring. For example, data of saira fishing expedition in the Pacific Ocean focuses on a set of background conditions:

• pronounced seasonal thermocline (0.19 deg/m or more);
• pronounced top quasi-homogeneous layer (6 m or more);
• presence of areas in the top quasi-homogeneous layer with the concentration of zooplankton of not less than 0.4 g/m^3^ and phytoplankton-5.6 g/m^3^.

Practical guides [4] assess such sites as the most favorable for fishing.

This example shows that the estimate is based on the combination of biotic and abiotic factors. Besides, the integral characteristic (concentration) of plankton as an estimate of the feed base plays a crucial role here.

The works [5,6,7] show why plankton studies are important within the ecosystem monitoring of the aquatic environment. A sustainable observation system requires measurements at large spatial scales and with long time series. For this purpose, it is proposed to use existing international [8,9,10] coordinated sampling programs and implemented infrastructure through additional measurements that could be carried out without significant changes in the infrastructure.

At the same time, it is possible to monitor biodiversity, thus obtaining information on individual plankton organisms with their species classification, which will further clarify the results of integral ecosystem measurements and, moreover, will provide information on the state of the ecosystem, the ecological situation in the water area, the presence and nature of other particles (suspensions of non-living particles, oil drops, gas bubbles, etc.).

International organizations recommend the active use of accompanying measurements for these purposes [11]. Accompanying measurements represent a practice that becomes common in operational oceanography, along with traditional measurements [12], which may also be implemented during background surveys, for example, in Argo, COPEPOD, and other projects.

Below there are several possible sensor carriers used to obtain data:

• Fishing vessels constantly present in the ocean. Despite the fact that the commercial interests of fishermen do not motivate them to oceanographic activities, mutually beneficial cooperation may be based on the principle of “accurate forecast in exchange for marine data”. An example of this approach to fisheries is RECOPESCA [13,14], where fishing vessels are voluntarily equipped with small sensors that record data on average catches and physical parameters, such as temperature or salinity. Information modules installed on board vessels automatically transmit data to the ground base station and the relevant monitoring center;
• Distributed ocean monitoring systems with measurement instruments for environmental diagnostics located near hazardous economic facilities [15]; These may include burial sites, industrial facilities, large ferry services, nuclear power stations, oil platforms, gas pipelines, etc.;
• Research vessels (RV) carrying out systematic studies of background conditions for obtaining and using data related to the natural world [16,17].

### Sensor Requirements for Accompanying Measurements

It is obvious that additional measurements require small-sized sensors with the possibility of fast transmission of measured data in the network mode to be placed on ships for accompanying measurements, as well as on existing infrastructure facilities (for example, Argo buoys). The overall characteristics of the sensors when placed on small AUVs (Autonomous Underwater Vehicle) [18,19], in particular gliders, are especially critical. At the same time, these sensors should measure the following background data for biodiversity and ecosystem monitoring:

• plankton concentration;
• plankton concentration by major taxa;
• average size and size dispersion of individuals;
• particle size distribution;
• average size of individuals and size dispersion within major taxa;
• particle size distribution within major taxa;
• water turbidity (turbidimetric parameter);
• suspension statistics (histogram by non-living particle size);
• parameters characterizing the vital activity of test organisms.

The list of measured parameters may be set in software by pre-setting or remotely depending on the current task. For example, the concentration of plankton in combination with size determines the biomass (as a food resource for fish), the average size and dispersion may characterize the aboriginal-invading species ratio within the taxon, while the size dispersion characterizes life cycle. The study of behavioral activity and technical possibilities for this are discussed in the work [20].

To describe plankton particles within taxa it is necessary to ensure their automatic recognition. Usually [21] we use eight main taxa for ecosystem classification: Chaetognatha, Phytoplankton colony, Phytoplankton chain, Copepoda, Copelata, Rotifera, Cladocera, and Other. This composition of plankton is chosen based on their greatest prevalence in the world ocean. From this perspective, the above refers to dominant and competing species. Their balance indicates the environmental sustainability of biocenosis.

The measurements should be representative; therefore, large medium volumes should be studied. However, it is quite difficult to simultaneously find a small sensor and a large size of the studied volume per one exposure. Hence, the sensor software should provide for the integration of information from adjacent measurement volumes.

Reliable and unambiguous interpretation of the obtained data requires information on the state of the habitat (temperature, salinity, oxygen saturation, etc.). Therefore, the sensor should be easily switched with standard hydrophysical sensors, including those located on existing infrastructure facilities.

Devices using fluorimetric, nephelometric, and turbidimetric measurements are used as instruments for studying marine particles (including plankton) in the oceanography [22]. However, the registration of plankton and other particles with the help of such equipment does not allow the study of individual particles and their classification. In this work, we propose using digital holography to study plankton. The advantage of digital holographic cameras in comparison with photographic cameras [23,24,25,26] lies in the registration in one exposure of information about all particles in the investigated volume. This makes it possible to obtain a sharp image of each of the particles located in the recorded volume from one hologram, and the numerical processing of particle images allows determination the geometric parameters of the particles (shape, size, position in space) and classifies them. Currently, there are both commercial submersible holographic cameras providing measurements of individual particles [27,28,29,30] and experimental cameras developed by research groups [31,32,33,34]. A detailed review of holographic cameras and their comparative analysis is presented in several works [35,36,37,38].

## 2. Materials and Methods

The previous experience of building digital holographic cameras (DHCs) was used to create a device for accompanying ecosystem measurements of plankton [35,39,40].

**Figure 1:**
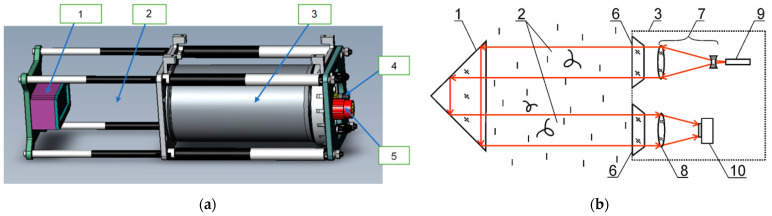
MiniDHC for accompanying measurements: (**a**) layout, (**b**) optical scheme. 1—mirror–prism system; 2—measured volume; 3—sealed case; 4—power connector; 5—Ethernet connector; 6—window; 7—beam expander; 8—receiving lens; 9—laser diode; 10—CMOS camera.

The model of a digital holographic camera considered here was called the miniDHC. Figure 1a shows the layout of the device; its technical characteristics are given in Table 1.

Figure 1b shows the optical scheme of the miniDHC representing an in-line holographic scheme in folded configuration.

The DHC principle of operation is based on the use of holography as a noninvasive method of recording and reconstruction of an image of the medium volume with particles, which makes it possible to characterize each particle suspended in the recorded studied volume. Since the hologram is recorded in a digital format, layer-by-layer reconstruction of the image of the studied volume is carried out numerically using on-board or ship computer [41,42,43]. In other words, the DHC technology (the mathematical processing) creates a virtual 3D image of the volume with analyzed particles [41].

The DHC technology allows not only registering and saving a hologram, but also restoring the spatial distribution of particles (three-dimensional coordinates of each particle), determining the size, shape, speed, and direction of movement of each particle and providing their recognition.

**Figure 2:**
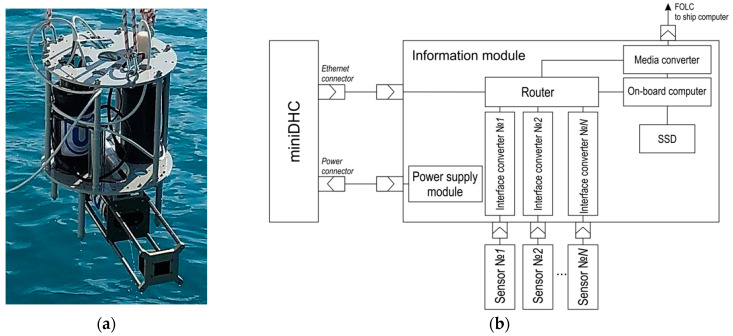
(**a**) MiniDHC as part of the hydrobiological probe. (**b**) Block diagram of the miniDHC connection, additional sensors and information module.

The miniDHC may operate as part of a hydrobiological probe (Figure 2a) supplied with an information module and a set of sensors. Figure 2b shows the block diagram of the hydrobiological probe. The need to use a media converter is determined by the selected type of FOCL (fiber optic communication line) communication with the ship computer, as well as the connection mode.

Unlike standard hydrophysical sensors, the computational task of the plankton sensor requires high-performance computing and a 1GB communication channel. Currently, there are no standard technical tools in compact submersible design that have the required performance to solve previously set measurement tasks (with the existing level of computer equipment). Therefore, in the considered miniDHC option such functions of the DHC technology as hologram recording, image reconstruction, and information processing are divided between the on-board and ship computers. This required the above-mentioned 1GB communication channel to transmit digital holograms to a high-performance ship computer.

Vertical sensing (measuring a vertical parameter profiles) of the marine environment in the practice of oceanographic measurements is most often carried out in one of the three ways:

1. A buoy with variable buoyancy is used. In this case, measurements are made without the ability to control data recording and processing parameters in real time. Autonomous on-board power is used. This option of power supply, as well as the methods of communication, information reading, and constructing a route for the considered case are presented in line No. 1, Table 2. The same mode may be applied for AUV with Wi-Fi or radio frequency data transmission when, for example, a glider is surfacing.
2. A hydrobiological probe is submerged from a shipboard (including the accompanying one), equipped with a standard winch with a paired SPC cable. Thus, the previous scenario is realized, but with ship power and the possibility of laying specified routes (line No. 2, Table 2);
3. A hydrobiological probe is submerged from a shipboard equipped with a standard winch with a FOCL SPC cable. This ensures real-time data processing (line No. 3, Table 2).

The second and third cases imply vertical sensing using an underwater hydrobiological probe with a vertical speed of not more than 1.0 m/s and a submersion depth of up to 500 m (the most representative layer for plankton study).

**Figure 3:**
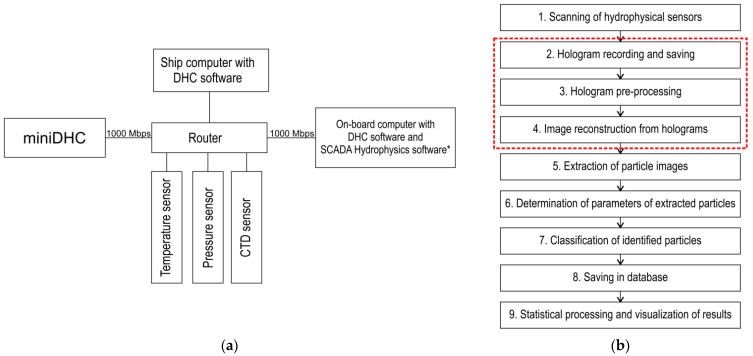
(**a**) Structure of interaction between hydrobiological probe devices, ship computer, and their software. *-optional. (**b**) Structure of the DHC software. The DHC basic software is marked red.

The general scheme of interaction of hydrobiological probe devices, ship computer and software is given in Figure 3. In general, all devices are connected to a local area network. This ensures flexible configuration of a probe as any computer is able to receive data from any sensor. Depending on the chosen design of the hydrobiological probe and the current task, the sequence of operations performed by devices and software may vary. For example, for options 1 and 2 (Table 2) the most efficient sequence of operations is as follows:

1. Launch of the DHC software on the on-board computer, probe submersion, implementation of items 1 and 2, Figure 3b (depending on the task, partial implementation of item 3 is possible);
2. If it is necessary to back up the hydrophysical data, the hydrophysics software, which works with SCADA support (Supervision Control And Data Acquisition) of ZETVIEW system, is launched before submersion [44];
3. To transmit data to the ship computer after lifting the probe, a Wi-Fi network or direct cable connection is used;
4. Data processing (items 3–7, Figure 3b) using parallel calculation algorithms and architectures is performed on the ship computer.

For option 3 in Table 2, the interaction scheme of the local area network elements is simplified, since control commands may be given from a ship computer, and moreover, digital holograms may be transmitted to the ship computer via FOCL and processed in real time (as far as the performance of the ship computer allows). At the same time, digital holograms are continuously recorded at the selected frame rate (up to 24 fps), and data processing and acquisition occurs once every 20 s. This ensures the discreteness of samples (6 m, Table 1) generated in real time at a submersion speed of the miniDHC equal to 0.3 m/s. Once the digital holograms are recorded, processing continues and a full depth profile is formed upon completion of processing.

Figure 3b shows the basic DHC software with red dotted line. Blocks 1, 5, 6, 7 may be changed and supplemented. For example, block 1 focuses on a set of available hydrophysical sensors. Blocks 5–7 may be modified and supplemented depending on the problem to be solved [40], for example, by a block for particle size distribution, forming video according to holographic data, determining particle speed, etc.

To present data on certain particles of the medium and their belonging to different taxa, the software includes a particle recognition and classification block (Figure 3b).

To implement the automatic classification method, a rectangle is circumscribed around the reconstructed holographic image of a particle. The classification uses the length of this rectangle H and the morphological parameter M, which is the ratio of the width and length of the rectangle, as well as the parameter defining the presence of antennas [41].

The characteristic values (decision tree) for automatic particle classification are shown in Table 3. The values of taxonomic features obtained through the analysis of plankton images from marine species identification databases [45,46] are tested on plankton images reconstructed from digital holograms and are given in [47].

As the experience of applying the automatic classification [21,35,39,40] has shown, the decision tree may be changed, for example, a new feature may be added-compactness [48], which may be expressed by the ratio of the area to the length of the particle image boundary. Such a possibility may be useful for more detailed or accurate classification, for example to diagnose gas hydrate methane bubbles or oil droplets.

As mentioned above, we usually use eight main taxa for ecosystem classification: Chaetognatha, Phytoplankton chain, Copepoda, Copelata, Rotifera, Cladocera, Other, and Marine snow. This list of taxa is chosen based on the greatest prevalence in the world ocean. Taxa 1–7 in Table 3 refer to plankton. Table 3 contains one more taxon-taxon 9 (Suspension), which refers to sedimentary particles and is used in this work to determine the turbidity of water.

Thus, the capabilities listed and commented on in this section, as well as features and parameters show that the developed miniDHC sensor has all the characteristics and capabilities indicated in the section “Sensor requirements for accompanying measurements”, and may be used for both special background and accompanying measurements.

## 3. Results and Discussion

**Figure 4:**
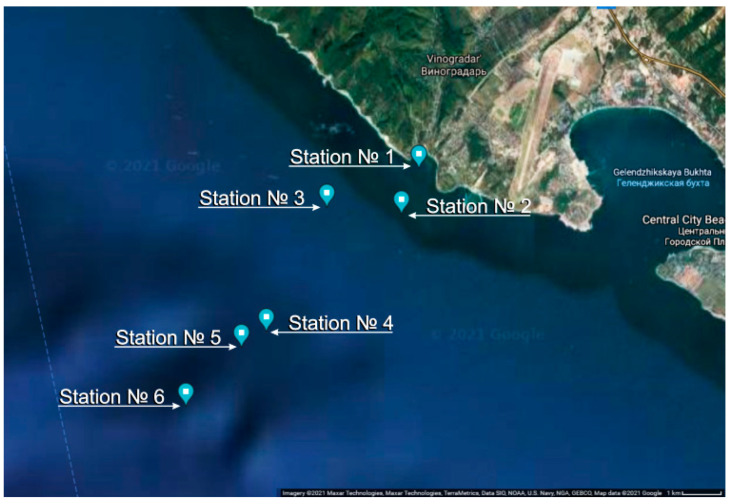
Studied water area and location of the stations: station No. 1 with coordinates 44°34.3623′ N 37°58.7232′ E, submersion up to 7 m (25 June 2019); station No. 2—44°33.7943′ N 37°58.4186′ E, up to 10 m (25 June 2019); station No. 3—44°33.8755′ N 37°57,1170′ E, up to 50 m (14 October 2019); station No. 4—44°32.1922′ N 37°56,0360′ E, up to 70 m (14 October 2019); station No. 5—44°32.0743′ N 37°55,3762′ E, up to 100 m (16 October 2019); station No. 6—44°31.2398′ N 37°54,3828′ E, up to 500 m (16 October 2019).

Field measurements to test the possibilities of the miniDHC took place at the Black Sea, near the city of Gelendzhik (Figure 4) from the end of June to mid-October 2019. Ashamba research vessel was used to perform accompanying measurements.

**Figure 5:**
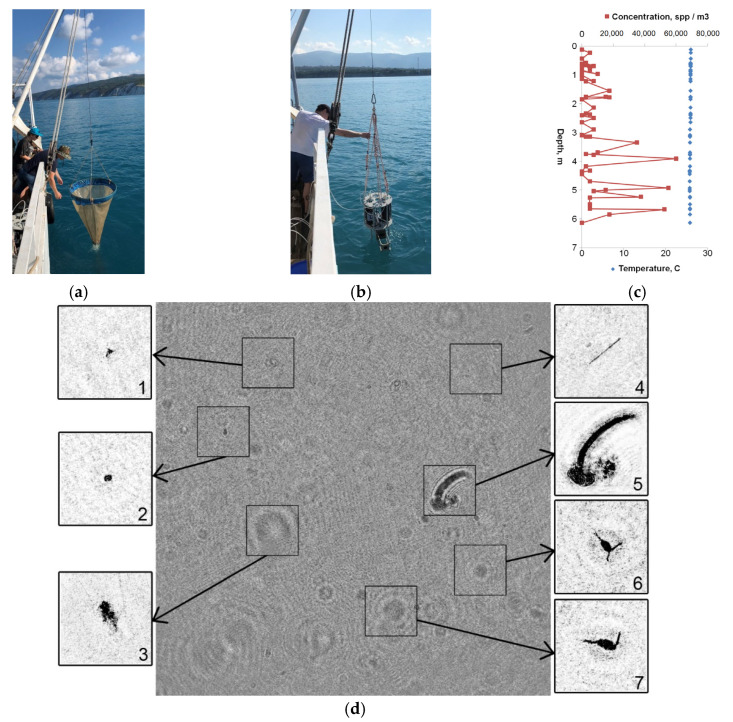
Equipment lowering in the Blue Bay on 25.06.19 from Ashamba research vessel: (**a**) Traditional plankton fishing using a net; (**b**) Hydrobiological probe with the miniDHC; (**c**) Profile of plankton concentrations (red) and one of the hydrophysical parameters (blue-temperature) recorded in situ in the photic layer and in real time. Measurements were made at station No. 1 with coordinates 44°34.3623′ N 37°58.7232′ E. (**d**) Digital hologram recorded at a depth of 1.8 m and reconstructed particle images: 1, 2—Suspension; 3—Marine snow; 4—Phytoplankton chain; 5—Copelata; 6, 7—Copepoda.

Figure 5 shows that the procedure for taking measurements using the miniDHC (Figure 5b) is similar to the standard procedure for taking plankton samples using a net (Figure 5a), with the only difference that the samples are virtual, and the result is obtained in real time (Figure 5c). The time of obtaining the plankton concentration plot point (Figure 5c) on the ship computer did not exceed 20 s, the full profile of plankton concentration to a depth of 7 m was formed within 17 min.

The measurement results in the post-experimental data processing mode on board the research vessel are recorded as the EXCEL table, which shows the parameters of all particles detected on a hologram and identified in accordance with the accepted taxonomy.

The required sampling of data on plankton particles and required recorded volume of the medium needed for representative results during statistical processing is estimated in works [39,49]. The required real-time scale for studied plankton processes was estimated in work [39].

**Figure 6:**
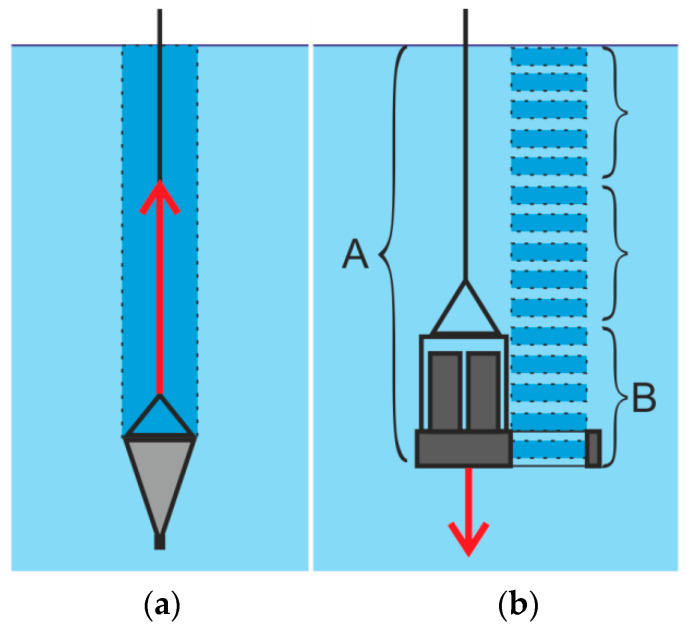
Sampling of particle data. The arrow shows the direction of movement to form a general sampling (**a**) Using a net, (**b**) Using the miniDHC. (A—sampled population; B—sampling from the sampled population to form one count).

Figure 6 shows the organization of measuring counts and data sampling using the plankton net (Figure 6a) and the miniDHC (Figure 6b).

According to this scheme, only the population generated sampling may be obtained using a net. At the same time, the miniDHC firmware allows flexibly controlling the virtual sampling mode. For example, for the case shown in Figure 6b, to form a single count (one point in a graph similar to Figure 5c), the volume data is averaged as 0.5 × 5 = 2.5 l. This function of flexible control of the virtual sampling mode provides additional opportunities for assessing the measurement error and controlling the representation of the studied volume of the aquatic medium.

From October 14 to 16, 2019, a series of stations was realized when the option No. 2 (Table 2) was implemented with WI-FI reading during the lifting with the research vessel winch and data processing in post-experimental mode, but with on-board battery power.

The results of the study in the form of plankton concentration by taxa obtained through automatic processing with the miniDHC are shown in Table 4. For comparison, the same table shows data obtained by the traditional method, i.e., by catching using a net, fixing and subsequent classification and counting in the manual mode under a microscope by an operator.

The data obtained by the miniDHC (averaging at the same maximum depth—7 m) differ from the data obtained by the traditional method by not more than:

6%—according to the total concentration of zooplankton individuals,
23%—Cladocera concentration,
11%—Copepoda concentration,
11%—Other taxon concentration.

However, the time that the miniDHC (17 min) takes for processing in the automatic mode is significantly less than the time in the manual mode (about a day, taking into account the time required to fix individuals). At the same time, the miniDHC makes it possible to obtain data in real time with regard to time scale of processes happening to plankton [39].

**Figure 7:**
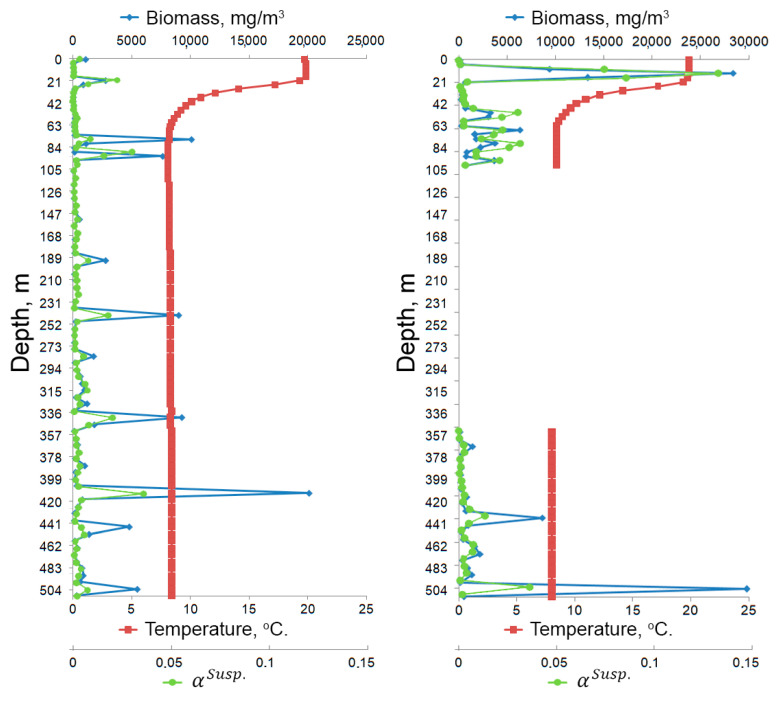
Example of background data represented by depth profiles. Sailing of Ashamba research vessel to the Black Sea on 16 October 2019, station No. 6 with coordinates 44°31.2398′ N 37°54.3828′ E, submersion up to 500 m: green graph—average turbidity-total fraction of the sectional area volume shielded by particle sections associated with the suspension taxon ($\alpha^{Susp.}$); red—water temperature; blue—average biomass of particles. (**a**) In forward motion (submersion); (**b**) In backward motion (lifting), in key areas.

**Figure 8:**
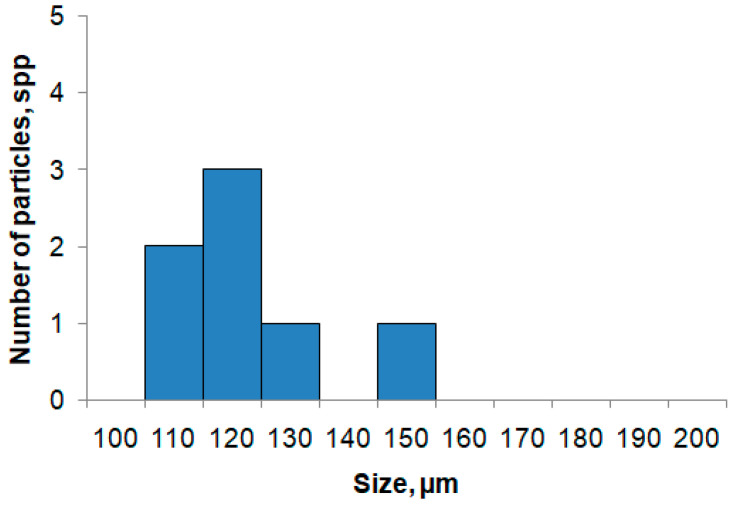
Example of background data on histogram formation according to the particle size of the Suspension taxon. Sailing of Ashamba research vessel to the Black Sea on 14 October 2019, station No. 3 with coordinates 44°33.8755′ N 37°57.1170′ E, submersion up to 50 m.

As a rule, turbidimetric and sediment measurements are performed to clarify the hydrological state of the water area (for example, turbidity measurements). The data obtained by the miniDHC also make it possible to assess turbidity and form a histogram by particle size of the suspension taxon. Turbidity is taken equal to the total part of the volume cross-sectional area shielded by particle sections-α. To calculate $\alpha^{k}$ for k taxon particles, the sectional area $S_{i}^{k}$ of the I particle is used

(1) $$\alpha^{k}=\frac{\sum_{i=0}^{n}S_{i}^{k}}{S_{0}^{p}}$$

where $S_{0}^{p}—$area of the miniDHC entrance pupil, n—number of particles of the *k* taxon. The total α (turbidity) of all particles is the sum of $\alpha^{k}$ particles of individual taxa. Figure 7 shows a diagram of the average $\alpha^{Susp.}$ Value associated with the suspension taxon. Figure 8 shows the distribution statistics of suspension size.

The literature sources [50,51] provide various methods to determine plankton biomass, including according to plankton images [52,53]. The miniDHC software may be adapted to the requirements of the selected biomass counting technique. This study determines the dimensions H (length circumscribed near the rectangle particle) of each particle suspended in water, i.e., a particle being in its raw state. In this case, the plankton density of any taxon may be taken equal to water density: $g\approx 1\;\mathrm{mg}/{\mathrm{mm}}^{3}$ [54]. To estimate the biomass by raw weight, each plankton particle is represented as an ellipsoid, the volume of which equals

(2) V=4πabc3=|c=H2,M=bc,a≈b=HM2|=16π·H3·M2

where a,b,c-semi-axes of the ellipsoid circumscribed around the particle, M—morphological parameter, H—particle size (length of the rectangle or ellipsoid circumscribed near the particle). The biomass of k taxon by raw weight is

(3) $$Biomass_{k}=\frac{\sum_{i=0}^{n}WW_{i}^{k}}{V_{0}}$$

where $WW_{i}^{k}$ = gVik=16π·Hik3·Mik2—raw weight (mg) of *I* particle in the measurement volume of taxon k, $V_{0}—$test volume of the DHC. The total biomass of all particles (Figure 7) is the sum of the biomass of individual taxa.

The data in Figure 7 show that the DHC processing technology allows calculating the measurement errors of sea particles and performing averaging according to a given volume. For example, at a depth of 21 m (Figure 7a), the average biomass of particles is 2770 ± 954 mg/m^3^, and the average value of $\alpha^{Susp.}$ is 0.02 ± 0.01 at a significance level of *p* = 0.05. The average data are presented as mean ± SD.

The given error regarding plankton characteristics measured using the miniDHC (including by classification) is high, but it does not go beyond the error found in oceanographic practice (~50%) [55]. The reasons for this error mainly concern the a priori choice of the medium volume (5 l) used for averaging in one count.

Systematic observations in oceanology are important not only because they provide for the formation of long series, but also because previous experience makes it possible to plan the next experiment more rationally. Our measurements did not have such a background, so not all parameters were chosen optimally.

The temperature graph (Figure 7) shows the thermocline located at a depth of 20–50 m, the temperature difference is 12 °C (from 20 to 8 °C). The differences in forward and backward schedules may be explained by increased turbulence when lifting the probe. This means that in order to obtain reliable data it is necessary to take measurements when submerging the miniDHC, as shown in Figure 6.

**Figure 9:**
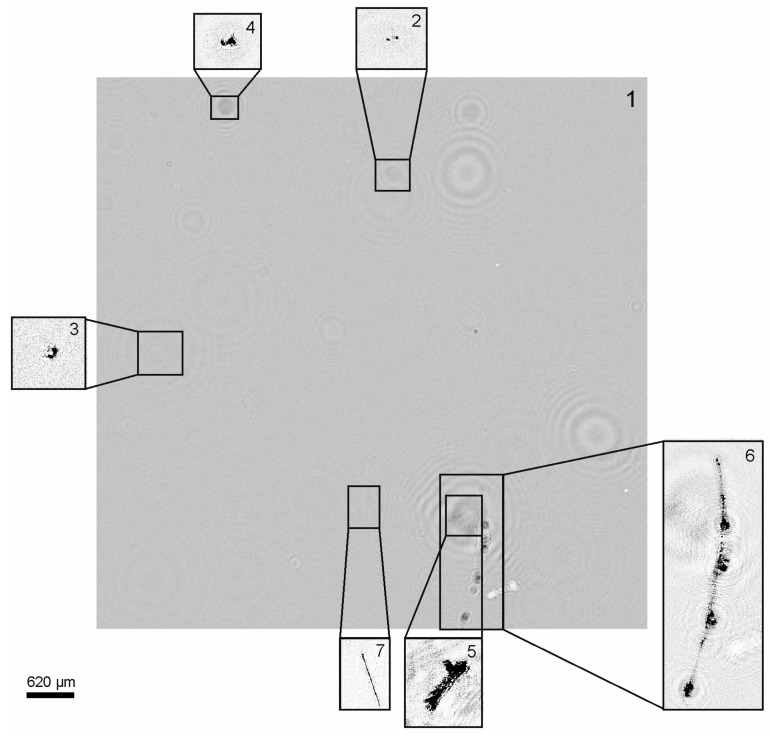
1—Digital hologram recorded at a depth of 388 m after removal of the background noise; 2, 3—reconstructed images of particles classified as ‘Other’; 4, 5, 6—reconstructed images of particles classified as ‘Marine snow’; 7—reconstructed image of a particle classified as ‘Phytoplankton chain’. The specified scale refers to reconstructed images.

The biomass distribution graph (Figure 7a) shows that the biomass values at a depth from 200 to 500 m are different from zero. The particle distribution by taxa showed that most of the particles in this mesopelagic zone are ‘Marine snow’, which drops from the surface layer deep [56,57,58]. Figure 9 shows the images of particles reconstructed from a digital hologram recorded at a depth of 388 m (as is known, in the Black Sea the hydrogen sulfide layer begins at a depth of about 200 m [59]). Three particles are classified as ‘Marine snow’, one as ‘Other’, and one as ‘Phytoplankton chain’.

**Figure 10:**
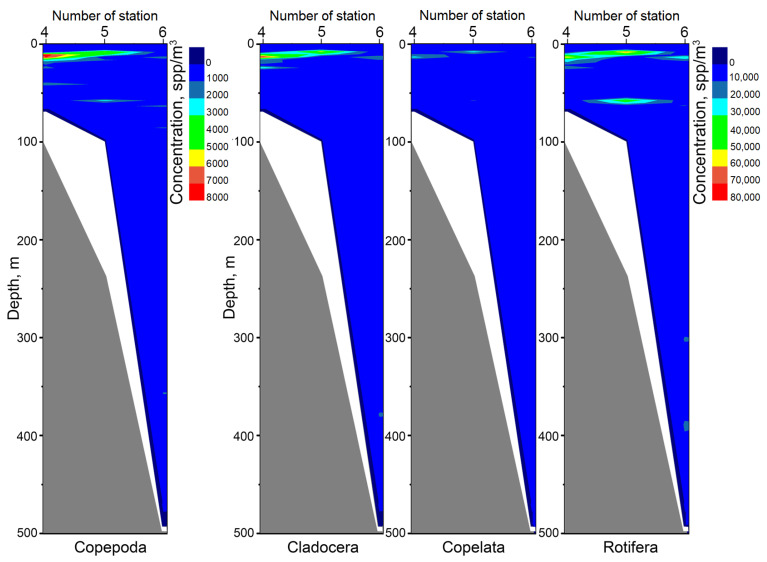
Average concentration profiles of particles related to four taxa (Copepoda, Cladocera, Copelata, Rotifera) over depth, measured by the miniDHC in forward motion (submersion) at stations: No. 4 (coordinates 044°32.1922′ N 37°56.0360′ E; submersion 0 up to 70 m), No. 5 (44°32.0743′ N 37°55.3762′ E; up to 100 m), No. 6 (44°31.2398′ N 37°54.3828′ E; up to 500 m). Gray shows the seabed profile, white (the absence of data (registration at this depth was not made). The right concentration scale refers to the graphs of Cladocera, Copelata, and Rotifera taxa, the left scale refers to Copepoda taxon.

Figure 10 shows the data for three stations with the submersion depth of 70 m (station No. 4), 100 m (station No. 5) and 500 m (station No. 6), in the forward motion (submersion) of the hydrobiological probe, obtained through postprocessing. Figure 10 shows the average particle concentration of the four taxa (Copepoda, Cladocera, Copelata, and Rotifera) after statistical averaging over 5 L. Color (blue to red) indicates the belonging of the average concentration at a given point to a certain range of values, gray shows the seabed profile, white indicates the absence of data (recording at this depth was not made). The stations were not further than 2 miles apart (Figure 4), which corresponds to the standard grid of stations for oceanographic background measurements. Interpolated data of plankton concentration is shown between stations. The image design was created using the OriginPro program, which uses linear interpolation when creating paths [60]. These results show that from the point of view of the ecosystem all significant changes in the plankton community occur up to a depth of 100 m, and almost regardless of the seabed profile. High concentration of plankton here is caused by the feed base of the photic layer. The proximity of thermocline contributing to the movement of organisms is also important. Here, as in Figure 7, it is located at a depth of 20–50 m, the temperature difference is 12 °C (from 20 to 8 °C). For example, for ecological monitoring in the ‘standby’ mode, i.e., in conditions of constant moorage, or for immediate diagnostics, we may limit ourselves to the thermocline area to compare monitoring data (study their dynamics). In this case, it is necessary to take into account the parameters of the thermocline (depth of occurrence, angle), the position of which is naturally determined by the season.

**Figure 11:**
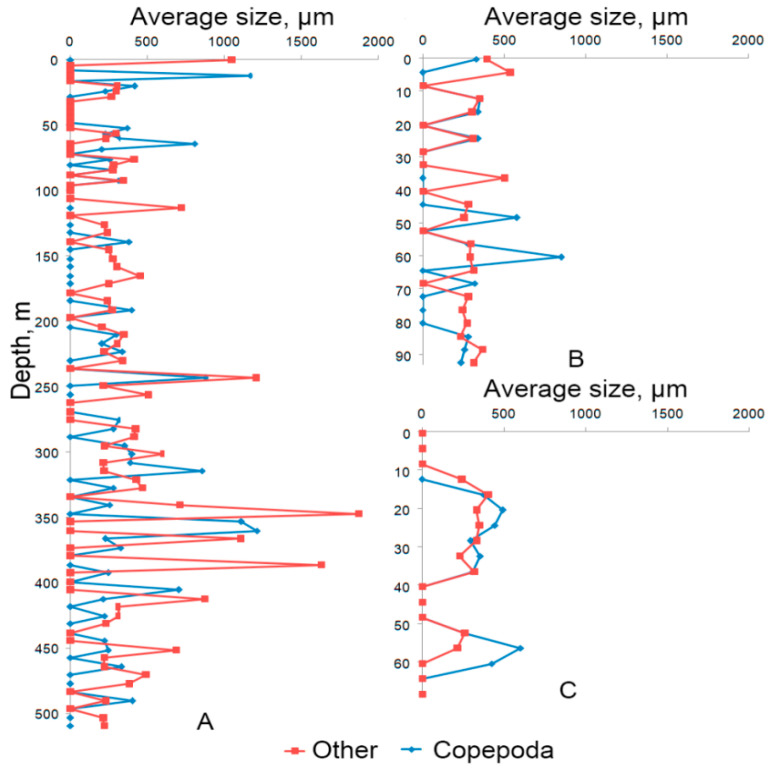
Average particle size by stations: (**A**) Station No. 6 with submersion up to 500 m and geographical coordinates 44°31.2398′ N 37°54.3828′ E; (**B**) Station No. 5 with submersion up to 100 m and coordinates 44°32.0743′ N 37°55.3762′ E; (**C**) Station No. 4 with submersion up to 70 m and coordinates 44°32.1922′ N 37°56.0360′ E.

**Figure 12:**
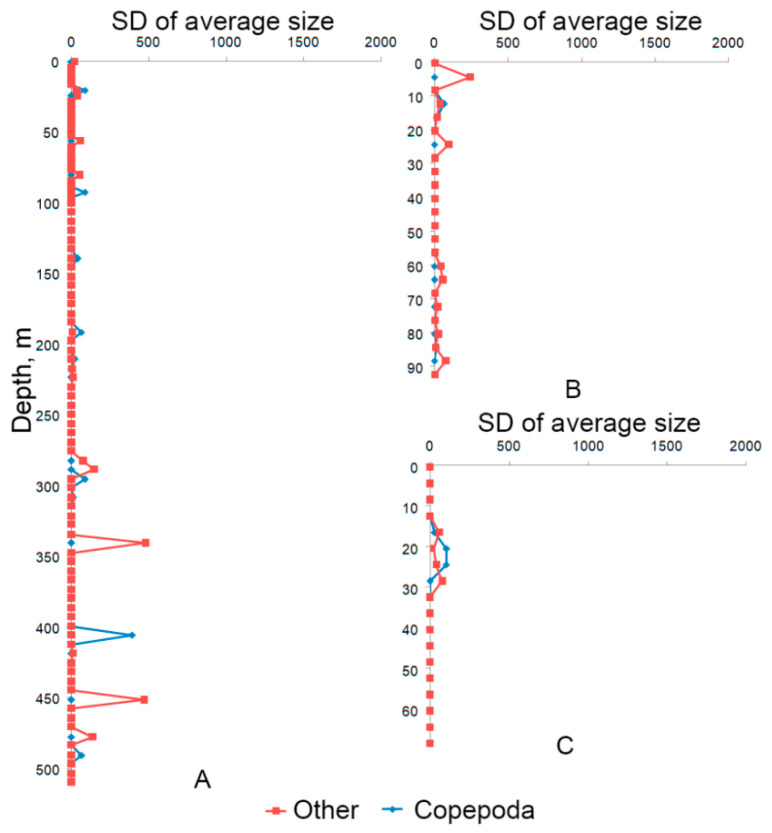
Standard deviation (SD) of particle sizes by stations: (**A**) Station No. 6 with submersion up to 500 m and geographical coordinates 44°31.2398′ N 37°54.3828′ E; (**B**) Station No. 5 with submersion up to 100 m and coordinates 44°32.0743′ N 37°55.3762′ E; (**C**) Station No. 4 with submersion up to 70 m and coordinates 44°32.1922′ N 37°56.0360′ E.

Figure 11 and Figure 12 show the profiles of the average particle size and the mean square deviation of particle size for the same stations with the submersion depth of 500, 100, and 70 m. The profiles are obtained as a result of data processing concerning forward probe motion (submersion). For example, the average particle size of Copepoda at a depth of 21 m at station No. 6 (submersion depth up to 500 m) was 419 ± 90 μm, ‘Other’-308 ± 33 μm. The average particle size of ‘Phytoplankton chain’ at a depth of 165 m was 146 ± 27 μm.

It should be noted that some graphs in Figure 7, Figure 11 and Figure 12 are not smooth (have jumps). This is caused by the a priori choice of the medium volume (5 l) used for averaging in one count (the same reason is indicated above when explaining the high random error estimate). To obtain smoother and more accurate curves, it is necessary to automate the selection of the averaging volume to ensure automatic regularization of data.

## 4. Conclusions

The field testing of the miniDHC showed that its capabilities correspond to the set of measurable background data required for biodiversity monitoring and ecosystem monitoring, such as:

• plankton concentration;
• plankton concentration by major taxa;
• average size and size dispersion of individuals;
• particle size distribution;
• average size of individuals and size dispersion within major taxa;
• particle size distribution within major taxa;
• water turbidity (calculated as the total fraction of the cross-sectional area of volume overlapped by the sections of the particles);
• suspension statistics (histogram by non-living particle size).

This paper does not consider the possibilities and features of the miniDHC for analyzing the parameters that characterize the vital activity of organisms. The study of behavioral activity and technical capabilities for this are discussed in the work [20].

The field tests of the miniDHC simulating accompanying measurements showed the compliance of the miniDHC specifications with the requirements for sensors for accompanying measurements:

• small dimensions (length—320.5 mm; diameter—142 mm; weight—9 kg);
• large analyzed volume per one exposure—0.5 l, in the count formation mode in 1 m—5 l;
• possibility to calculate measurement errors of marine particle parameters and perform averaging according to the specified volume;
• capability of combining with standard hydrophysical sensors (temperature sensor, pressure sensor, etc.).

The comparison of the concentration data of individual taxa obtained by the miniDHC with the data obtained by the traditional method of catching the plankton using a net showed a difference of not more than:

• 6%—total concentration of zooplankton,
• 23%—Cladocera concentration,
• 11%—Copepoda concentration,
• 11%—Other taxon concentration.

In this case, the time that the miniDHC (17 min) takes for processing in the automatic mode is significantly less than the time in the manual mode (about a day, taking into account the time required to register individuals).

It is also worth noting the obvious non-invasiveness of in-situ studies conducted with the help of the miniDHC.

The characteristics of the holographic camera presented above and confirmed by field tests indicate that the miniDHC is promising for accompanying (background) measurements.

To further improve the device characteristics, which were identified during the described offshore operations and discussed in this article, the following studies are assumed:

• adding the particle compactness parameter to the number of features for classification
• introduction of elements of adaptive control of the sample size of experimental data in the formation of counts for plankton
• comparison of the results of suspension turbidimetry with alternative measurement methods.

## 5. Patents

Dyomin V.V., Polovtsev I.G., Olshukov A.S. The method of registration of plankton. The patent of the Russian Federation No. 623984 dated 31.08.2016.
Dyomin V.V., Davydova A.Yu., Kirillov N.S., Olshukov A.S., Polovtsev I.G. The method of recording the integral size-quantitative characteristics of plankton. The patent of the Russian Federation No. 2690976 dated 09.11.2018.
Dyomin V.V., Olshukov A.S., Davydova A.Yu., Kirillov N.S. DHC-Plankton V1.2. Certificate of state registration of computer programs No. 2019667359 dated 23.12.2019.

## Figures and Tables

**Table 1:** MiniDHC specifications.

Parameter	Value
Power characteristics:	
– power voltage, V	12
– power consumption, W	20
Volume studied per one exposure, l	0.5
Averaging volume, l	5
Working volume length, mm	338.4
Submersion depth, m, not more than	500
Size of measured particles, mm	0.1–28
Submersion speed, m/s	0.1–1.0
Discreteness of counts formed in real time at the submersion speed of 0.3 m/s, m	6
Ethernet transmission rate, GB/s	1
Hydrostatic pressure tolerance, atm	60
Overall dimensions (length × diameter), mm	320.5 × 142
Weight, kg, not more than	9

**Table 2:** Options for hydrobiological probe design with the miniDHC.

Approach	Measurement Management Link	Data Transmission to a Ship	Power Supply	On-Board Computer	Visual Information on Ship Computer	Route
Measurements with autonomous on-board power supply	-	WI-FI+ twisted pair	Battery or carrier power (buoy, glider, etc.)	Available, carrier computer (buoy, glider, etc.) may be used	-	Random, determined by the carrier motion algorithm (buoy, glider, etc.)
Ship-fed measurements	-	WI-FI+ twisted pair	Battery, ship’s SPC cable	Available	-	Route of an accompanying ship with a winch
Real-time measurement and processing	FOCL SPC cable 500 m or more	FOCL	Ship’s SPC cable	Available	Possible in real time	Route of an accompanying ship with a winch with FOCL SPC cable

**Table 3:** Characteristic values of taxa for automatic classification of particles: M—morphological parameter; H—length of a rectangle circumscribed around the particle image.

Taxa	Presence of Antennas	H, µm	M
1. Chaetognatha	YES	>200	0–0.2
2. Copepoda	YES	>200	0.2–0.5
3. Copelata	YES	>200	0.5–0.66
4. Cladocera	YES	>200	0.66–0.9
5. Other	YES	>200	0.9–1
6. Rotifera	YES	≤200	0–0.9
7. Phytoplankton chain	NO	ANY	0–0.25
8. Marine snow	NO	ANY	0.25–0.9
9. Suspension	NO	≤200	0.9–1

**Table 4:** Marine data for comparison of plankton measurements with average at the same maximum depth 7 m.

	Traditional Classification of Plankton Sample Using a Net		Plankton Classification Using the miniDHC	
n/n	Organism	Concentration, spp/m^3^	Taxon	Concentration, spp/m^3^
1	*Sagitta setosa* < 10 mm	7.05	Chaetognatha	0
2	Copepoda < 1 mm	430.53	Copepoda	720.33
3	*Nauplii Copepoda*	38.95		
4	*Acartia clausi*	0.84		
5	*Centropages kroyeri*	307.37		
6	*Oithona davisae*	34.74		
7	Harpacticoida	0.63		
8	*Oikopleura dioica*	0.32	Appendicularia	0
9	*Plepois polyphemoides*	54.74	Cladocera	42.37
10	*Noctiluca miliaris*	0.11	Other	400.84
11	*Larvae Gastropoda*	326.32		
12	*Larvae Bivalvia*	0.32		
13	*Larvae Polychaeta*	1.05		
14	*Larvae Decapoda*	1.37		
15	*Nauplii Cirripedia*	42.11		
16	*Cypris* st., *Ostracoda*	0.11		
17	Ova Fish	1.79		
18	Actinotrocha	0.21		
	Total	1248.53		1163.54

## Data Availability

The data presented in this study are openly available in Zenodo at https://doi.org/10.5281/zenodo.4866468 (accessed on 16 July 2021).
